# Treating patients with platinum-sensitive extensive-stage small-cell lung cancer in a real-world setting

**DOI:** 10.3389/fonc.2023.1161931

**Published:** 2023-12-22

**Authors:** Jacob Sands, Janakiraman Subramanian

**Affiliations:** ^1^ Thoracic Oncology, Dana-Farber Cancer Institute, Boston, MA, United States; ^2^ Division of Oncology, Saint Luke’s Cancer Institute, Kansas City, MO, United States; ^3^ Center for Precision Oncology, Saint Luke’s Cancer Institute, Kansas City, MO, United States

**Keywords:** small-cell lung cancer, SCLC, chemotherapy, radiotherapy, immunotherapy, immune checkpoint inhibitors, platinum resistant, platinum sensitive

## Abstract

Extensive-stage small-cell lung cancer (ES-SCLC) is an aggressive disease with poor 5-year survival. The first-line standard-of-care for ES-SCLC is platinum plus etoposide, along with 1 of the immune checkpoint inhibitors atezolizumab or durvalumab. Although SCLC first-line therapy often leads to rapid responses, treatment becomes more challenging at progression, particularly for those with a chemotherapy-free interval (CTFI) of ≤6 months. The NCCN Clinical Practice Guidelines in Oncology (NCCN Guidelines^®^) for SCLC no longer specify treatment recommendations in this setting, but options approved by the US Food and Drug Administration include topotecan and lurbinectedin. Participation in a clinical trial is recommended as an option regardless of CTFI. Other NCCN-recommended regimens are paclitaxel, irinotecan, temozolomide, and cyclophosphamide/doxorubicin/vincristine, among others. Nivolumab and pembrolizumab are options in those not previously treated with a checkpoint inhibitor. For patients with platinum-sensitive SCLC (CTFI >6 months), preferred treatment per the NCCN Guidelines^®^ for SCLC is retreatment with platinum and etoposide, although the use of immune checkpoint inhibitors is discouraged if there is progression on a drug in this class. Further research on immunotherapies and combination regimens is ongoing, and continuing work on the subcharacterization of SCLC may lead to better precision of therapies that promote more durable responses in individual patients with ES-SCLC.

## Introduction

Small-cell lung cancer (SCLC) is a high-grade neuroendocrine tumor strongly associated with a significant smoking history and represents ~13% of all lung cancer cases (1). In the United States alone, an estimated 33,006 patients were diagnosed with SCLC in 2021, with a 5-year survival rate of only 7% (1).

Staging of SCLC is often categorized in clinical practice as limited stage (LS) or extensive stage (ES) (2); approximately 70% present with ES-SCLC (3). SCLC is aggressive regardless of the disease stage at diagnosis. While responses to initial therapy are common (2, 3), aggressive and resistant disease at the time of progression results in median survival of 12-13 months for patients with ES-SCLC (3–5).

In this review, we describe our experiences in the diagnosis and treatment of SCLC, focusing on regimens within the NCCN Clinical Practice Guidelines in Oncology (NCCN Guidelines^®^) for SCLC we consider most useful in the second-line setting and beyond.

## Diagnosis and staging of SCLC

SCLC diagnosis is generally made based on a hematoxylin and eosin stain and classic immunohistochemical stains (6, 7). Dual inactivation of 2 tumor suppressors, p53 (*TP53*) and RB (*RB1*), is present in most cases (8, 9). Although genomic testing is not typically part of the workup, molecular profiling is recommended for patients without a smoking history in ES-SCLC (7). The most prominent staging systems are the Veterans Administration (VA) and Tumor, Node, Metastasis (TNM) classification systems (7, 10). The National Comprehensive Cancer Network^®^ (NCCN^®^) describes TNM staging within a VA description, including a subcategorization within LS-SCLC (7).

The VA classification system is a 2-stage scheme that defines LS-SCLC as confined to the ipsilateral hemithorax and safely encompassed within a radiation field (7, 10). ES-SCLC is defined as disease present beyond the ipsilateral hemithorax, including malignant pleural or pericardial effusion, or hematogenous metastases (7, 10). The VA classification system is most commonly used for clinical decision making, although the TNM system may assist in further categorization of LS-SCLC, as highlighted by the NCCN Guidelines^®^ for SCLC (7, 10, 11). For example, the TNM system can be useful in selecting patients with T1-2, N0 disease who are eligible for surgery and radiation (7).

Full staging of SCLC includes a history and physical examination, computed tomography (CT) scan of the chest, abdomen, pelvis, and brain imaging with magnetic resonance imaging (MRI) or CT scan if MRI cannot be performed (7). Scanning with positron emission tomography (PET) is recommended in LS-SCLC (7). Lesions detected by PET/CT that would result in upstaging should be confirmed by pathologic examination if not considered definitive by imaging alone (7).

## First-line treatment of patients with ES-SCLC

### Chemoimmunotherapy

According to the NCCN Guidelines for SCLC (V.3.2023), the preferred first-line regimen for patients with LS-SCLC is platinum plus etoposide with concurrent radiotherapy (7). For patients with ES-SCLC, the NCCN recommends platinum plus etoposide with either atezolizumab or durvalumab (7) for first-line therapy based on overall survival (OS) results from the IMpower133 and CASPIAN phase 3 trials, respectively (4, 5).

In IMpower133, patients with previously untreated ES-SCLC were randomized 1:1 to atezolizumab (carboplatin/etoposide and atezolizumab) or placebo (carboplatin/etoposide and placebo). Treatment led to a median OS benefit of 12.3 versus 10.3 months, respectively (hazard ratio [HR]=0.76 [95% confidence interval (CI): 0.60-0.95]; *P*=0.007); addition of atezolizumab also resulted in improved OS at 1 year (51.9% vs 39.0%) and at 18 months (34.0% vs 21.0%) (12).

In CASPIAN, patients with ES-SCLC were randomized 1:1:1 to receive first-line treatment with platinum-etoposide plus durvalumab, platinum-etoposide plus durvalumab and tremelimumab, or platinum-etoposide alone (5). Patients in the durvalumab group had significantly longer median OS than platinum-etoposide alone (13.0 vs 10.3 months; HR=0.73 [95% CI: 0.59-0.91]; *P*=0.0047) (5). Tremelimumab was not associated with improvement in OS beyond that seen in the control group (13). A 3-year update showed continued OS benefit with durvalumab versus platinum-etoposide alone (HR=0.71 [95% CI: 0.60-0.86]), with 3 times as many patients alive at 3 years in the durvalumab group (17.6% vs 5.8%) (14).

Other immunotherapies have been tested for use as first-line treatment, including pembrolizumab and nivolumab (15, 16), which were previously given accelerated approval by the US Food and Drug Administration (FDA) for the treatment of metastatic SCLC in later-line settings (17, 18). In the KEYNOTE-604 trial, first-line treatment with pembrolizumab plus platinum-etoposide was associated with significant prolongation of progression-free survival (PFS) versus placebo plus platinum-etoposide (12-month PFS estimate: 13.6% vs 3.1%; HR=0.75 [95% CI: 0.61-0.91]; *P*=0.0023), but pembrolizumab just missed the significance boundary for OS (15). When considered along with the results of CheckMate 331, in which nivolumab in the second-line setting did not outperform chemotherapy (19), applications for full FDA approval were withdrawn (20, 21). It is important to note, however, the potential benefit that still exists from single-agent nivolumab or pembrolizumab in the first-line setting, particularly in the durability of responses.

Although not registrational, in the EA5161 trial of first-line ES-SCLC, nivolumab plus platinum-etoposide showed statistically significant improvement in median PFS versus platinum-etoposide alone (5.5 vs 4.6 months; HR=0.65 [95% CI: 0.46-0.91]; *P*=0.012); the secondary endpoint of median OS was also improved with nivolumab (11.3 vs 8.5 months; HR=0.67 [95% CI: 0.46-0.98]; *P*=0.038) *(* 16).

## Supportive care for the management of chemotherapy-induced myelosuppression

Standard treatments for ES-SCLC often result in some degree of chemotherapy-induced myelosuppression (22). According to the NCCN Guidelines, granulocyte colony-stimulating factor (G-CSF) or trilaciclib may be used prophylactically to decrease the incidence of chemotherapy-induced myelosuppression when treating with particular regimens (7). G-CSF has demonstrated reduced neutropenia and febrile neutropenia compared with placebo when given with chemotherapy (cyclophosphamide, doxorubicin, and etoposide) for SCLC (23). Although the regimen in the study is no longer used, G-CSF is an accepted adjunct and utilized in patients considered to be at higher risk for complications of neutropenia. G-CSF has not demonstrated any impact on cancer treatment outcomes such as response rate or survival (24).

Pooled data from 3 randomized, double-blind, placebo-controlled trials (NCT02499770, NCT03041311, and NCT0251447) evaluating the effects of trilaciclib versus placebo when administered prior to chemotherapy found that trilaciclib was associated with significant decreases in most measures of myelosuppression and improvement in health-related quality of life (HRQoL) metrics (22, 25–27). These trials did not allow for primary prophylaxis with G-CSF. Many patients do not require prophylaxis, but we usually consider G-CSF for cases when concerned about neutropenia and consider trilaciclib only for patients at risk for prolonged anemia or thrombocytopenia. The HRQoL metrics noted to be improved with trilaciclib, such as anemia, highlight the potential for greater impact of chemotherapy-induced anemia than is often clinically appreciated.

## Second- and later-line treatment of patients with platinum-sensitive SCLC

Platinum sensitivity or resistance is often defined differently among clinical trials, as shown in **Table 1** (28, 30, 31, 33–35). FDA-approvals of topotecan and lurbinectedin are defined by the enrollment criteria of the respective trials (32, 36). Although definitive cutoff times for chemotherapy-free interval (CTFI) exist, platinum sensitivity should be considered a continuum in clinical practice (28).

**Table 1:** Definitions of Platinum-sensitive and Platinum-resistant Disease in Relapsed SCLC.*

Reference	Platinum sensitive	Platinum resistant
Lara 2015 (28)	CTFI ≥90 days	CTFI <90 days
NCCN 2022 (7)	CTFI >6 months	CTFI ≤6 months
ESMO 2021 (29)	CTFI ≥3 months	CTFI <3 months
Oral topotecan PI (30)	Approved for relapsed SCLC and CTFI ≥45 days	**—**
IV topotecan PI (31)	Approved for relapsed SCLC and CTFI ≥60 days	**—**
Trigo 2020 (32)	CTFI ≥90 days	CTFI <90 days

CTFI, chemotherapy-free interval; ESMO, European Society for Medical Oncology; IV, intravenous; NCCN, National Comprehensive Cancer Network; PI, prescribing information; SCLC, small-cell lung cancer.

*Based on time until progression after last platinum dose.

The likelihood of response to treatment in the second-line setting and beyond decreases, and prognosis worsens (8, 37). However, a longer CTFI increases the likelihood of a clinically meaningful response to other cytotoxic agents (2).

### NCCN-recommended subsequent systemic therapies

According to the NCCN Guidelines, rechallenge with the original regimen or similar platinum-based regimen (without immune checkpoint inhibitors [ICI] if previously given) is the preferred regimen in patients with a CTFI >6 months and may also be considered in patients with a CTFI 3-6 months (7). Retreatment with platinum-based therapy has been a long-standing standard of care, originating at a time of substantially fewer options. A more recent comparison to topotecan as second-line therapy in patients with at least a 3-month CTFI demonstrated an improvement in PFS with platinum-etoposide retreatment but no significant difference in OS (38). Inclusion of a clinical trial as recommended therapy highlights the ongoing need for improved options in this setting. Topotecan and lurbinectedin are the FDA-approved options after progression on first-line platinum-based therapy (30, 31, 36). Other regimens are described by NCCN (7); we consider the most relevant to be paclitaxel, irinotecan, and temozolomide. We consider nivolumab and pembrolizumab to be important options only for patients not previously treated with ICI as part of first-line treatment (7). **Table 2** presents data on subsequent therapy options for ES-SCLC.

**Table 2:** Second-line Treatment Options for ES-SCLC.

Trial	SCLC population(s)	Administration schedule	Key efficacy outcomes	Key safety outcomes/concerns	Overall conclusions	Key management considerations (7)*
Topotecan PO or IV						
von Pawel *J Clin Oncol* 1999 (33)						
Topotecan	Randomized trial; LS- or ES-SCLC; progression ≥60 days after first-line chemo	IV topotecan 1.5 mg/m^2^/d for 5 days every 21 days (n=107)	• ORR 24.3% • TTP 13.3 weeks • DoR 14.4 weeks • Median OS 25.0 weeks • Significantly greater improvement with topotecan for several symptoms	• Grade 3/4 leukopenia 87% • Grade 3/4 neutropenia 88% • Grade 3/4 thrombocytopenia 58% • Grade 3/4 anemia 42%	• Efficacy of topotecan similar to that of CAV, but topotecan showed a better toxicity profile • Topotecan more effective in controlling several symptoms	Topotecan IV is FDA approved as a second-line regimen if CTFI is ≥60 days
CAV	Randomized trial; LS- or ES-SCLC; progression ≥60 days after first-line chemo	CAV (cyclophosphamide 1000 mg/m^2^, doxorubicin 45 mg/m^2^, and vincristine 2 mg) on Day 1 every 21 days (n=104)	• ORR 18.3% • TTP 12.3 weeks • DoR 15.3 weeks • Median OS 24.7 weeks	• Grade 3/4 leukopenia 81% • Grade 3/4 neutropenia 87% • Grade 3/4 thrombocytopenia 15% • Grade 3/4 anemia 20%	• Efficacy of CAV similar to that of topotecan	CAV is an NCCN “other recommended” regimen
O’Brien *J Clin Oncol* 2006 (34)	Phase 3 trial; relapsed SCLC; ≥45 days after first-line chemo	Randomized 1:1 to PO topotecan 2.3 mg/m^2^/d on Days 1-5 every 21 days plus BSC (n=71) or BSC alone (n=70)	• Median OS 25.9 vs 13.9 weeks • ORR to topotecan 7%; another 44% with stabilization of disease • TTP 16.3 weeks	• Grade 3/4 neutropenia with topotecan 61% • Grade 3/4 thrombocytopenia with topotecan 38% • Grade 3/4 anemia with topotecan 25% • All-cause mortality within 30 days, 7% topotecan vs 13% BSC	PO topotecan prolonged survival and improved QoL compared with BSC alone	Topotecan PO is FDA as a second-line regimen if CTFI ≥45 days
Eckardt *J Clin Oncol* 2007 (35)	Phase 3, open-label trial; relapsed SCLC; ≥90 days after first-line chemo	Randomized 1:1 to PO topotecan 2.3 mg/m^2^/d (n=153) or IV topotecan 1.5 mg/m^2^/d (n=151) on Days 1-5 every 21 days	• ORR 18.3% PO vs 21.9% IV • DoR 18.3 vs 25.4 weeks • Median OS 33.0 vs 35.0 weeks • TTP 11.9 vs 14.6 weeks	• Grade 4 leukopenia 23% vs 26% • Grade 4 neutropenia 47% vs 64% • Grade 4 thrombocytopenia 29% vs 18% • Grade 3 or 4 anemia in 23% vs 31%	Similar antitumor efficacy and safety/tolerability profiles of PO and IV topotecan	Topotecan IV is FDA approved as a second-line regimen if CTFI is ≥60 days; topotecan PO is FDA approved as a second-line regimen if CTFI is ≥45 days
Lurbinectedin						
Trigo *Lancet Oncol* 2020 (32)	Single-arm, open-label, phase 2, basket trial; only 1 previous chemo regimen	• Lurbinectedin 3.2 mg/m^2^ infusion over 1 hour every 3 weeks (n=105) until disease progression or unacceptable toxicity • Median follow-up 17.1 months	• ORR 35.2% • DoR 5.3 months • PFS 3.5 months • Median OS 9.3 months	Most common grade 3/4 AEs and lab abnormalities: • Anemia 9% • Leukopenia 29% • Neutropenia 46% • Thrombocytopenia 7% • Febrile neutropenia 5%	Good efficacy and acceptable and manageable safety profile	Lurbinectedin is FDA approved regardless of CTFI
Subbiah *Lung Cancer* 2020 (39)	Phase 2 study; candidates for platinum rechallenge; CTFI ≥180 days	• Lurbinectedin 3.2 mg/m^2^ infusion over 1 hour every 3 weeks (n=20) until disease progression or unacceptable toxicity • Median follow-up 15.6 months	• ORR 60.0% • DoR 5.5 months • PFS 4.6 months • Median OS 16.2 months	Most common grade 3/4 AEs and lab abnormalities: • Neutropenia 55% • Anemia 10% • Thrombocytopenia 10% • Fatigue 10% • Increased liver function tests 5%-10%	Good efficacy for platinum-sensitive relapsed SCLC, especially with CTFI ≥180 days, with acceptable safety and tolerability	Lurbinectedin is FDA approved regardless of CTFI
Paclitaxel						
Smith *Br J Cancer* 1998 (40)	Phase 2 study; relapsed SCLC; <3 months after last chemo	Paclitaxel 175 mg/m^2^ infusion over 3 hours every 21 days (n=24)	• ORR 29% • DoR 3.6 months • TTP 2.1 months • Median OS 3.3 months	• Grade 3/4 leukopenia: 10 of 63 evaluable cycles • Grade 3/4 thrombocytopenia: 5 of 63 cycles • 2 early deaths and 1 death due to toxicity (12.5%)	Modest efficacy (but low enrollment population)	Paclitaxel is an NCCN “other recommended” regimen
Yamamoto *Anticancer Res* 2006 (41)	Phase 2 study; relapsed or refractory SCLC; <1 month after last chemo	Paclitaxel 80 mg/m^2^ infusion over 1 hour for 6 weeks, then 2 weeks without treatment (8-week cycle; n=21)	• ORR 23.8% • Median OS 5.8 months	• Grade 3/4 leukopenia 48% • Grade 3/4 neutropenia 67% • Death 4.8%	Modest efficacy (but low enrollment population)	Paclitaxel is an NCCN “other recommended” regimen
**Docetaxel** Smyth *Eur J Cancer* 1994 (42)	Phase 2 study; metastatic or locally advanced SCLC; with or without prior chemo	Docetaxel 100 mg/m^2^ infusion over 1 hour every 21 days (n=34)	• ORR 25% • DoR 4.7 months	• Grade 3/4 leukopenia 69% • Grade 3/4 neutropenia 94% • Grade 3 anemia 5% (no grade 4)	Modest efficacy; high numbers of cytopenias	Docetaxel is an NCCN “other recommended” regimen
**Irinotecan** Masuda *J Clin Oncol* 1992 (43)	Phase 2, nonrandomized study; relapsed or refractory SCLC; ≥1 month after last chemo	Irinotecan 100 mg/m^2^ infusion over 90 minutes every week (n=15)	• ORR 47% • DoR 1.9 months	• Grade ≥3 leukopenia 33% • Grade ≥3 anemia 20%	High response rate (but low numbers) in heavily pretreated patients	Irinotecan is an NCCN “other recommended” regimen
Temozolomide						
Pietanza *Clin Cancer Res* 2012 (44)	Phase 2, open-label study; platinum-sensitive (≥60 days after first-line chemo) or refractory (<60 days after first-line chemo) SCLC	Temozolomide 75 mg/m^2^/d PO on Days 1-21 of a 28-day cycle (n=64; n=48 sensitive; n=16 refractory)	• ORR 20% (23% sensitive cohort; 13% refractory cohort) • DoR 3.5 months • Median OS 5.8 months (sensitive 6.0 months; refractory 5.6 months) • TTP 1.6 months (sensitive 1.6 months; refractory 1.0 month)	• Grade 3/4 leukopenia 3% • Grade 3/4 neutropenia 5% • Grade 3/4 thrombocytopenia 9% • Grade 3/4 lymphopenia 30% • Grade 3 anemia 3% (no grade 4)	Sufficient response rate for consideration, but may benefit from better patient selection and potentially from combination therapy	Temozolomide is an NCCN “other recommended” regimen
Zauderer *Lung Cancer* 2014 (45)	Platinum-sensitive (≥60 days after first-line chemo) or refractory (<60 days after first-line chemo) SCLC	Temozolomide 200 mg/m^2^/d PO on Days 1-5 of each 28-day cycle (n=25; n=16 sensitive; n=9 refractory)	• ORR 12% • TTP 1.8 months • Median OS 5.8 months	• Grade 3/4 toxicity in 5 patients (anemia, thrombocytopenia, neutropenia, and constipation) • Grade 3/4 thrombocytopenia 16% • Grade 3/4 lymphopenia 76%	Primary endpoint of AEs was met, with grade 3/4 toxicity (excluding lymphopenia) in only 5 patients	Temozolomide is an NCCN “other recommended” regimen
PO etoposide						
Johnson *J Clin Oncol* 1990 (46)	Phase 2 study; relapsed or refractory SCLC; ≥3 weeks after last chemo (≥1 previous chemo regimen)	Etoposide 50 mg/m^2^/d PO for 21 consecutive days (n=22)	• ORR 45.5% • DoR 4.0 months • Median OS 3.5+ months (range, 1.0-15.0+ months)	• Dominant toxicity was myelosuppression • Life-threatening leukopenia in 18% of cycles • Severe thrombocytopenia in 25% of cycles	PO etoposide shows good activity for recurrent SCLC in patients with response to previous treatment and CTFI ≥90 days	PO etoposide is an NCCN “other recommended” regimen
Vinorelbine						
Janssem *Eur J Cancer* 1993 (47)	Phase 2 study; relapsed SCLC; ≥3 months after first-line chemo	Vinorelbine 30 mg/m^2^ infusion over 20 minutes weekly (n=25)	• ORR 16%	• Leukopenia 80% (32% grade 3/4) • Neutropenia 72% (32% grade 3/4)	Limited response rate; leukopenia was the only limiting toxicity	Vinorelbine is an NCCN “other recommended” regimen
Furuse *Oncology* 1996 (48)	Phase 2 study; relapsed or refractory SCLC; >1 month after last chemo	Vinorelbine 25 mg/m^2^ infusion weekly (n=24)	• ORR 12.5% • DoR in the 3 responders was 56, 64, and 99 days	• Leukopenia 92% (67% grade 3/4) • Neutropenia 88% (71% grade 3/4) • Anemia 71% (21% grade 3/4)	Low response rate in patients resistant to multiple therapies; leukopenia was the major toxicity	Vinorelbine is an NCCN “other recommended” regimen
Gemcitabine						
van der Lee *Ann Oncol* 2001 (49)	Relapsed LS- or ES-SCLC; progression <3 months after last chemo	Gemcitabine 1000 mg/m^2^ infusion over 30 minutes on Days 1, 8, and 15 of a 28-day cycle (n=38)	• ORR 13% • DoR 10-20 weeks • Median OS 17 weeks • TTP 8 weeks	• Grade 3 thrombocytopenia 29% (no grade 4) • Grade 3 leukopenia 18% (no grade 4)	Limited response rate	Gemcitabine is an NCCN “other recommended” regimen
Masters *J Clin Oncol* 2003 (50)	Phase 2 study; progressive LS- or ES-SCLC; platinum-sensitive (≥90 days after first-line chemo) or refractory (<90 days after first-line chemo)	Gemcitabine 1000 mg/m^2^ infusion over 30 minutes on Days 1, 8, and 15 of a 28-day cycle (n=42)	• ORR 11.9% • DoR 1.8-4.1 months • Median OS 7.1 months	• Grade 3/4 leukopenia 18% • Grade 3/4 neutropenia 27% • Grade 3/4 thrombocytopenia 27% • Grade 3/4 anemia: 7%	Limited response rate; favorable toxicity profile	Gemcitabine is an NCCN “other recommended” regimen
Nivolumab						
Antonia *Lancet Oncol* 2016 (51)						
Nivolumab	Phase 1/2, open-label study; progressive LS- or ES-SCLC; platinum-sensitive (≥90 days after last chemo) or refractory (<90 days after last chemo)	Nivolumab 3 mg/kg infusion every 2 weeks until disease progression or unacceptable toxicity (n=98)	• ORR 10% • DoR not reached	Grade 3/4 treatment-related AEs: 13%	Clinically meaningful activity, durable response, and acceptable safety for SCLC that progressed after platinum-containing chemo	Nivolumab is an NCCN “other recommended” regimen; use is discouraged if there is progression on maintenance atezolizumab or durvalumab at time of relapse
Nivolumab + ipilimumab	Phase 1/2, open-label study; progressive LS- or ES-SCLC; platinum-sensitive (≥90 days after last chemo) or refractory (<90 days after last chemo)	Nivolumab plus ipilimumab infusion every 3 weeks for 4 cycles at 3 dose levels: • 1 + 1 mg/kg, n=3 • 1 + 3 mg/kg, n=61 • 3 + 1 mg/kg, n=54 All combinations followed by nivolumab 3 mg/kg every 2 weeks until disease progression or unacceptable toxicity	ORR: • 1 + 1 mg/kg 33% • 1 + 3 mg/kg 23% • 3 + 1 mg/kg 19% DoR: • 1 + 1 mg/kg not reached • 1 + 3 mg/kg 7.7 months • 3 + 1 mg/kg 4.4 months	Grade 3/4 treatment-related AEs: • 1 + 1 mg/kg 0 • 1 + 3 mg/kg 30% • 3 + 1 mg/kg 19% Deaths from treatment-related AEs: • 1 + 1 mg/kg, 0 • 1 + 3 mg/kg, n=2 • 3 + 1 mg/kg, n=1	Clinically meaningful activity, with durable responses; acceptable safety profile in single-arm study after progression on platinum-containing chemo	Nivolumab + ipilimumab is not specifically mentioned by NCCN as a second-line regimen
Ready *J Thorac Oncol* 2020 (52)						
Nivolumab	Update of a randomized cohort study; progressive LS- or ES-SCLC after 1 or 2 previous chemo regimens	Nivolumab 3 mg/kg infusion every 2 weeks until disease progression or unacceptable toxicity (n=147)	• ORR 11.6% • DoR 15.8 months • PFS 1.4 months • Median OS 5.7 months	Grade 3/4 treatment-related AEs 12.9%	Nivolumab monotherapy had lower ORR than nivolumab + ipilimumab but also lower toxicity	Nivolumab is an NCCN “other recommended” regimen; use is discouraged if there is progression on maintenance atezolizumab or durvalumab at time of relapse
Nivolumab + ipilimumab	Update of a randomized cohort study; progressive LS- or ES-SCLC after 1 or 2 previous chemo regimens	Nivolumab 1 mg/kg plus ipilimumab 3 mg/kg every 3 weeks for 4 cycles, followed by nivolumab 3 mg/kg every 2 weeks until disease progression or unacceptable toxicity (n=96)	• ORR 21.9% • DoR 10.0 months • PFS 1.5 months • Median OS 4.7 months	Grade 3/4 treatment-related AEs 37.5%	Nivolumab + ipilimumab improved ORR compared with nivolumab monotherapy but had increased toxicity; higher response rate did not translate into longer PFS or OS	Nivolumab + ipilimumab is not specifically mentioned by NCCN as a second-line regimen
Pembrolizumab						
Ott *J Clin Oncol* 2017 (53)	Phase 1b, open-label study; progressive ES-SCLC that expressed PD-L1	Pembrolizumab 10 mg/kg every 2 weeks for 2 years or until disease progression or unacceptable toxicity (n=24)	• ORR 33.3% • DoR 19.4 months • PFS 1.9 months	• Grade 3-5 AEs in 33.3% (treatment related in 2 patients)	Promising antitumor activity and durable response in pretreated patients positive for PD-L1; favorable safety profile	Pembrolizumab is an NCCN “other recommended” regimen; use is discouraged if there is progression on maintenance atezolizumab or durvalumab at time of relapse
Chung *J Clin Oncol* 2018 (54)	Phase 2 study; relapsed or refractory SCLC; evaluable for PD-L1	Pembrolizumab 200 mg once every 3 weeks for 2 years or until disease progression or unacceptable toxicity (n=107; PD-L1 positive n=42; PD-L1 negative n=50)	• ORR overall 18.7% (PD-L1 positive 35.7%; PD-L1 negative 6.0%) • DoR overall not reached • PFS overall 2.0 months • Median OS 9.1 months (PD-L1 positive 14.6 months; PD-L1 negative 7.7 months)	• Treatment-related AEs (grade not specified) 59% • 1 death	Limited response rate; median PFS limited due to low numbers of responders, but significant durability among responders; ORR and OS better in PD-L1–positive disease	Pembrolizumab is an NCCN “other recommended” regimen; use is discouraged if there is progression on maintenance atezolizumab or durvalumab at time of relapse
Chung *J Thorac Oncol* 2020 (55)	Pooled analysis of 2 trials; progressive SCLC after ≥2 lines of previous chemo; no previous immune checkpoint inhibitor therapy	Pembrolizumab 10 mg/kg every 2 weeks (KEYNOTE-028 study) or 200 mg every 3 weeks (KEYNOTE-158 study) for up to 2 years or until disease progression, unacceptable toxicity, or intercurrent illness (n=83)	• ORR 19.3% (response in 88% of PD-L1–positive patients) • PFS 2.0 months • DoR not reached • Median OS 7.7 months	• Grade 3-5 treatment-related AEs 9.6% • 2 deaths	Limited response rate but impressive durability among responders; good tolerability; supports use as a third-line therapy	Pembrolizumab is an NCCN “other recommended” regimen; use is discouraged if there is progression on maintenance atezolizumab or durvalumab at time of relapse
Bendamustine						
Lammers *J Thorac Oncol* 2014 (56)	Phase 2 study, relapsed SCLC, platinum-sensitive (≥90 days after last chemo) or refractory (<90 days after last chemo)	Bendamustine 120 mg/m^2^ infusion on Days 1 and 2 of a 21-day cycle (n=50)	• ORR 26% (sensitive 33%; resistant 17%) • TTP 4.0 months • Median OS 4.8 months (sensitive 5.7 months; resistant 4.1 months)	The most common grade 3/4 AEs: • Fatigue 20% • Dyspnea 12% • Anemia 12%	Only category 2B of NCCN Guidelines and not generally used	Bendamustine is an NCCN “other recommended” regimen (category 2B)

AE, adverse event; BSC, best supportive care; CAV, cyclophosphamide/doxorubicin/vincristine; CTFI, chemotherapy-free interval; DoR, duration of response; ES, extensive stage; FDA, US Food and Drug Administration; IV, intravenous; LS, limited stage; NCCN, National Comprehensive Cancer Network; ORR, overall response rate; OS, overall survival; PD-L1, programmed death ligand 1; PFS, progression-free survival; PO, oral; QoL, quality of life; SCLC, small-cell lung cancer; TTP, time to progression.

*All NCCN recommendations are category 2A unless otherwise indicated.

### Topotecan

Topotecan was the first drug approved for second-line treatment of relapsed SCLC, in 1996 (30, 31). Intravenous topotecan was approved based on a second-line trial including patients with a CTFI ≥60 days (31). Outcomes with topotecan 1.5 mg/m^2^/day were not significantly different from CAV (cyclophosphamide 1000 mg/m^2^, doxorubicin 45 mg/m^2^, and vincristine 2 mg) for median PFS (13.3 vs 12.3 weeks) or OS (25.0 vs 24.7 weeks), but topotecan was associated with significantly better symptomatic improvement (33).

Oral topotecan was approved for second-line SCLC after a CTFI ≥45 days (30) after a trial randomized patients who were not candidates for further intravenous chemotherapy to oral topotecan 2.3 mg/m^2^/day or best supportive care. A significantly longer median OS was noted with topotecan (25.9 vs 13.9 weeks; adjusted HR=0.61 [95% CI: 0.43-0.87]); measures of symptomatic improvement and HRQoL also favored topotecan (34).

A trial of patients randomized to oral topotecan 2.3 mg/m^2^/day versus intravenous topotecan 1.5 mg/m^2^/day as second-line therapy for SCLC (CTFI ≥90 days) found a similar overall response rate (ORR; 18.3% vs 21.9%) and median OS (33.0 vs 35.0 weeks) (35).

### Lurbinectedin

Lurbinectedin is the first drug to be approved for SCLC in the second-line setting since topotecan (32). Lurbinectedin monotherapy (3.2 mg/m^2^ every 3 weeks) was granted accelerated FDA approval in 2020 for adults with progressing metastatic SCLC after platinum-based chemotherapy (36). Accelerated approval was based on the primary endpoint of ORR (35.2% [95% CI: 26.2-45.2]; investigator assessed) and median duration of response (5.3 months [95% CI: 4.1-6.4]) from the SCLC cohort in a phase 2 basket trial (32, 36). Treatment was associated with a manageable safety profile, with grade 3-4 events most commonly cytopenias (32). Although neutropenia was common, neutropenic fever was noted in 5% of patients, and primary G-CSF prophylaxis was not allowed in the trial. In clinical practice, primary G-CSF prophylaxis may be considered, particularly for patients at higher risk for prolonged neutropenia or infection.

Among patients with a CTFI ≥6 months, Subbiah and colleagues reported on the 20-patient subset from the lurbinectedin monotherapy (3.2 mg/m^2^) basket trial (39). The subset achieved an ORR of 60.0% (95% CI: 36.1-86.9) and median OS of 16.2 months (95% CI: 9.6 to upper limit not reached) (39). Combined with the acceptable safety profile, the data suggest lurbinectedin is a favorable option in this setting (39). A phase 3 confirmatory trial (NCT05153239) in patients with relapsed second-line SCLC was initiated in 2021 (57, 58).

ATLANTIS, a randomized trial comparing combination lurbinectedin (2.0 mg/m^2^) and doxorubicin versus physician’s choice of topotecan or CAV following progression on one platinum-containing line did not meet its primary endpoint of significance for OS (59, 60). Nonetheless, the ATLANTIS trial showed a superior safety and tolerability profile for lurbinectedin-doxorubicin compared to the control arm, with significantly lower rates of hematologic toxicities (59, 60). It should be noted that the approved dose of lurbinectedin monotherapy is 3.2 mg/m^2^, as opposed to the lower dose used in combination therapy in the ATLANTIS trial. The confirmatory phase 3 study noted above includes a 3-arm design that will compare lurbinectedin as either monotherapy or in combination with irinotecan versus investigator’s choice of irinotecan or topotecan.

### Other NCCN-recommended options

Irinotecan and paclitaxel are common treatment options for SCLC despite lacking FDA approval. Data specific to SCLC efficacy for each are limited, but the side effect profile is well described as cornerstone treatments for other common cancer diagnoses (61–63). Irinotecan has shown responses with weekly dosing and is generally well tolerated, with diarrhea being a notable toxicity (43). We generally start with dosing 100 mg/m^2^ on Days 1 and 8 of a 21-day cycle. Paclitaxel can be dosed every 3 weeks or weekly and has similarly shown responses (40). We prefer weekly dosing (6 weekly doses of an 8-week cycle) due to the toxicity profile.

Temozolomide is another option included in the NCCN Guidelines that is worth noting due to excellent central nervous system (CNS) penetration, highlighted by its standard use for brain tumors such as glioblastoma (64). Brain metastases are common complications in SCLC and can be challenging to treat at recurrence after prior whole brain radiation. This setting, in particular, is one for consideration of temozolomide as a treatment.

After ≥2 prior lines of therapy, pembrolizumab as a single agent showed a median PFS of only 2.0 months but an ORR of 19%, with a durability beyond 18 months in >60% of responders (55). The durability of responses to single-agent pembrolizumab and nivolumab along with their tolerable side effect profiles (51, 52, 55) is why both immunotherapy agents are present in the NCCN recommendations for second-line treatment and beyond, but these are only options for patients not previously treated with an ICI (7).

### Practical considerations for the second-line treatment of platinum-sensitive SCLC following chemoimmunotherapy

Data on second-line and beyond treatments for patients with prior exposure to immunotherapy are limited; thus, second-line treatment options are not restricted based on prior immunotherapy. Individuals with progression on first-line chemoimmunotherapy are not candidates for subsequent immunotherapy treatment. For patients not treated with an ICI in the first-line setting, the role for single-agent ICI is debated but we feel should be considered in certain patients. This situation arises almost exclusively in patients who were treated for LS-SCLC.

If the time to recurrence is prolonged, combination platinum-etoposide and an ICI can be considered, but for patients with a shorter duration to recurrence or a contraindication to platinum-etoposide, we might consider treatment with an ICI alone based on the impressive durability noted when there is a response. A pooled analysis of 2 single-arm trials of pembrolizumab demonstrated an ORR of 19%, with more than half of responders experiencing ongoing disease control beyond 2 years (55). Similarly, nivolumab demonstrated a median duration of response of ~18 months (65). Ipilimumab in combination with nivolumab in the second-line setting did not demonstrate advantages over nivolumab alone and increased toxicity was noted, leading to removal of ipilimumab from the NCCN Guidelines (52).

## Case report

A 73-year-old woman with a 25 pack/year history of smoking was diagnosed with metastatic SCLC from a liver biopsy with radiographic findings, including a right-lung lower-lobe nodule, extensive bilateral hilar and mediastinal adenopathy, and liver metastases. Brain MRI was negative for metastasis. She received 4 cycles of carboplatin-etoposide and atezolizumab with good treatment response and, in shared discussion with radiation oncology, elected for MRI brain monitoring every 3 months without prophylactic cranial irradiation. Maintenance atezolizumab continued for an additional 7 cycles before progression was noted with multiple brain metastases and progressive liver lesions. She received whole brain radiation therapy (WBRT) followed by second-line treatment with single-agent lurbinectedin. Although platinum-based doublet is preferred by the NCCN in the setting of CTFI >6 months (rechallenge may also be considered for patients with CTFI 3-6 months), this patient chose lurbinectedin for the easier side effect profile and once every 3 weeks schedule. We do not regularly retreat with carboplatin-etoposide due to the diminishing durability of response with future therapy lines unless it was initially very well tolerated with a particularly prolonged CTFI. The patient did well on lurbinectedin, with shrinking liver metastases before progression at 6 months,which included growing liver metastases and new bone metastases.

In this setting, we usually choose irinotecan rather than topotecan due to the side effect profile and treatment schedule. Paclitaxel is also a favorable option. If the patient had not previously received ICI, we would consider nivolumab or pembrolizumab to be important considerations and generally try to initiate ICI in the setting of low tumor burden, if possible. Due to the limited responses to nivolumab and pembrolizumab as single agents, an opportunity for other treatment options may be lost in those with large tumor burden and/or symptoms, but duration of response and drug toxicity for ICI is favorable enough that these should be considered if no prior ICI has been received.

If brain metastases were noted as the site of progression after prior WBRT, temozolomide would have been our preference due to excellent CNS penetration. Topotecan is another option with CNS penetration in patients not previously treated with a topoisomerase I inhibitor.

Additionally, we prioritize clinical trial enrollment for most patients and recommend referral to a center with trials for patients being treated in a setting without local trial options.

## Unmet need and future therapies in the treatment of SCLC

Although therapeutic options are still limited for patients with ES-SCLC, preclinical and clinical studies are ongoing. Studies are being conducted on various immunotherapies that attempt to stimulate a stronger immune response and on antibody-drug conjugates deliver cytotoxic drugs with greater precision (66). Tarlatamab is a bispecific T-cell engager molecule that targets delta-like ligand 3 (DLL-3), as well as CD3 on T cells (66, 67). DLL-3 is selectively expressed on SCLC tumors, with little to no expression in healthy lung cells (66, 67). In preclinical studies, tarlatamab has shown good potency and specificity, promoting SCLC lysis even in cell lines with low DLL-3 expression (68). A phase 1 study (NCT03319940) investigating tarlatamab plus pembrolizumab in patients with relapsed/refractory SCLC is ongoing (67, 69), as is a monotherapy dose-ranging trial in relapsed/refractory SCLC (NCT05060016) (70). HPN328 is an anti–DLL-3 T-cell engager that directs T cells to DLL-3–expressing SCLC cells and initiates tumor cell lysis (71). A phase 1/2 study (NCT04471727) to assess the safety and pharmacokinetics of HPN328 in patients with advanced cancers that have failed standard therapy is currently open and recruiting (72).

Poly(ADP-ribose) polymerase inhibitors may have a role in the treatment of SCLC. A randomized, double-blind study of veliparib and temozolomide versus temozolomide plus placebo in relapsed/refractory SCLC showed no improvement in the primary endpoint, 4-month PFS (36% vs 27%; *P*=0.19), but the ORR was higher (39% vs 14%; *P*=0.016) (73). SLFN11 expression correlated with a significantly longer PFS and OS for the combination of veliparib and temozolomide, highlighting the potential as a biomarker pending further study (73). SLFN11 immunohistochemistry is being used to select patients for a phase 2 trial (NCT04334941) of atezolizumab plus talazoparib versus atezolizumab alone as maintenance therapy for ES-SCLC (74, 75).

Multiple other studies are ongoing for second-line and beyond SCLC (76, 77). Many challenges remain, but novel discoveries and improved diagnostics (including proposed subtypes) may lead to better selection for novel therapies, which could improve responses (78–82).

## Conclusions

ES-SCLC is an aggressive disease with poor 5-year survival. SCLC generally responds to first-line treatment with platinum-etoposide along with atezolizumab or durvalumab, but treatment in the second-line setting and beyond is more challenging. Topotecan was the only approved second-line treatment for 40 years, and lurbinectedin monotherapy was granted accelerated FDA approval in 2020 for the treatment of disease progression on or after platinum-based chemotherapy. The NCCN Guidelines for SCLC include various subsequent treatment options after progression on first-line treatment. We consider the most relevant to be the FDA-approved options, including topotecan and lurbinectedin, as well as paclitaxel, irinotecan, and temozolomide. Nivolumab or pembrolizumab are important considerations in patients not previously treated with ICI. Further understanding of SCLC subtypes has the potential to improve treatment selection, and enrollment in clinical trials continues to be an important treatment option for patients with SCLC.

## Data availability statement

The original contributions presented in the study are included in the article/supplementary material. Further inquiries can be directed to the corresponding author.

## Author contributions

JSa and JSu contributed to the writing, review, and editing of the manuscript. All authors contributed to the article and approved the submitted version.

## References

[1] American Cancer Society. Cancer facts & figures 2021 (2021). Available at: https://www.cancer.org/content/dam/cancer-org/research/cancer-facts-and-statistics/annual-cancer-facts-and-figures/2021/cancer-facts-and-figures-2021.pdf (Accessed Janaury 20, 2023).

[2] Bernhardt EB Jalal SI. Small cell lung cancer. Cancer Treat Res (2016) 170:301–22. doi: 10.1007/978-3-319-40389-2_14 27535400

[3] National Cancer Institute. Small cell lung cancer treatment (PDQ®)-health professional version (2022). Available at: https://www.cancer.gov/types/lung/hp/small-cell-lung-treatment-pdq (Accessed March 16, 2021).

[4] Horn L Mansfield AS Szczesna A Havel L Krzakowski M Hochmair MJ. First-line atezolizumab plus chemotherapy in extensive-stage small-cell lung cancer. N Engl J Med (2018) 379:2220–9. doi: 10.1056/NEJMoa1809064 30280641

[5] Paz-Ares L Dvorkin M Chen Y Reinmuth N Hotta K Trukhin D. Durvalumab plus platinum-etoposide versus platinum-etoposide in first-line treatment of extensive-stage small-cell lung cancer (CASPIAN): a randomised, controlled, open-label, phase 3 trial. Lancet (2019) 394:1929–39. doi: 10.1016/S0140-6736(19)32222-6 31590988

[6] Travis WD. Update on small cell carcinoma and its differentiation from squamous cell carcinoma and other non-small cell carcinomas. Mod Pathol (2012) 25(Suppl 1):S18–30. doi: 10.1038/modpathol.2011.150 22214967

[7] Referenced with permission from the NCCN Clinical Practice Guidelines in Oncology (NCCN Guidelines^®^) for small cell lung cancer V.3.2023 (2022). © National Comprehensive Cancer Network, Inc (Accessed February 3, 2023). To view the most recent and complete version of the guidelines, go online to NCCN.org. NCCN makes no warranties of any kind whatsoever regarding their content, use or application and disclaims any responsibility for their application or use in any way.

[8] Rudin CM Brambilla E Faivre-Finn C Sage J. Small-cell lung cancer. Nat Rev Dis Primers (2021) 7:3. doi: 10.1038/s41572-020-00235-0 33446664 PMC8177722

[9] George J Lim JS Jang SJ Cun Y Ozretic L Kong G. Comprehensive genomic profiles of small cell lung cancer. Nature (2015) 524:47–53. doi: 10.1038/nature14664 26168399 PMC4861069

[10] Kalemkerian GP Gadgeel SM. Modern staging of small cell lung cancer. J Natl Compr Canc Netw (2013) 11:99–104. doi: 10.6004/jnccn.2013.0012 23307985

[11] Vallieres E Shepherd FA Crowley J Van Houtte P Postmus PE Carney D. The IASLC Lung Cancer Staging Project: proposals regarding the relevance of TNM in the pathologic staging of small cell lung cancer in the forthcoming (seventh) edition of the TNM classification for lung cancer. J Thorac Oncol (2009) 4:1049–59. doi: 10.1097/JTO.0b013e3181b27799 19652623

[12] Liu SV Reck M Mansfield AS Mok T Scherpereel A Reinmuth N. Updated overall survival and PD-L1 subgroup analysis of patients with extensive-stage small-cell lung cancer treated with atezolizumab, carboplatin, and etoposide (IMpower133). J Clin Oncol (2021) 39:619–30. doi: 10.1200/JCO.20.01055 PMC8078320 33439693

[13] Goldman JW Dvorkin M Chen Y Reinmuth N Hotta K Trukhin D. Durvalumab, with or without tremelimumab, plus platinum-etoposide versus platinum-etoposide alone in first-line treatment of extensive-stage small-cell lung cancer (CASPIAN): updated results from a randomised, controlled, open-label, phase 3 trial. Lancet Oncol (2021) 22:51–65. doi: 10.1016/S1470-2045(20)30539-8 33285097

[14] Paz-Ares L Chen Y Reinmuth N Hotta K Trukhin D Statsenko G. Durvalumab ± tremelimumab + platinum-etoposide in first-line extensive-stage SCLC (ES-SCLC): 3-year overall survival update from the phase III CASPIAN study. Ann Oncol (2021) 32(suppl 5):S1283–346. doi: 10.1016/annonc/annonc741

[15] Rudin CM Awad MM Navarro A Gottfried M Peters S Csőszi T. Pembrolizumab or placebo plus etoposide and platinum as first-line therapy for extensive-stage small-cell lung cancer: randomized, double-blind, phase III KEYNOTE-604 Study. J Clin Oncol (2020) 38:2369–79. doi: 10.1200/JCO.20.00793 PMC7474472 32468956

[16] Leal T Wang Y Dowlati A Lewis DA Chen Y Mohindra AR. Randomized phase II clinical trial of cisplatin/carboplatin and etoposide (CE) alone or in combination with nivolumab as frontline therapy for extensive-stage small cell lung cancer (ES-SCLC): ECOG-ACRIN EA5161. J Clin Oncol (2020) 38:9000. doi: 10.1200/JCO.2020.38.15_suppl.9000 PMC12276847 40526078

[17] US Food and Drug Administration. FDA approves pembrolizumab for metastatic small cell lung cancer (2019). Available at: https://www.fda.gov/drugs/resources-information-approved-drugs/fda-approves-pembrolizumab-metastatic-small-cell-lung-cancer (Accessed January 13, 2022).

[18] US Food and Drug Administration. FDA grants nivolumab accelerated approval for third-line treatment of metastatic small cell lung cancer (2018). Available at: https://www.fda.gov/drugs/resources-information-approved-drugs/fda-grants-nivolumab-accelerated-approval-third-line-treatment-metastatic-small-cell-lung-cancer (Accessed January 13, 2022).

[19] Spigel DR Vicente D Ciuleanu TE Gettinger S Peters S Horn L. Second-line nivolumab in relapsed small-cell lung cancer: CheckMate 331^☆^ . Ann Oncol (2021) 32:631–41. doi: 10.1016/j.annonc.2021.01.071 33539946

[20] Merck. Merck provides update on KEYTRUDA® (pembrolizumab) indication in metastatic small cell lung cancer in the US (2021). Available at: https://www.businesswire.com/news/home/20210301005851/en/ (Accessed January 13, 2022).

[21] Bristol Myers Squibb. Bristol Myers Squibb statement on Opdivo (nivolumab) small cell lung cancer U.S. indication (2020). Available at: https://news.bms.com/news/details/2020/Bristol-Myers-Squibb-Statement-on-Opdivo-nivolumab-Small-Cell-Lung-Cancer-US-Indication/default.aspx.

[22] Weiss J Goldschmidt J Andric Z Dragnev KH Gwaltney C Skaltsa K. Effects of trilaciclib on chemotherapy-induced myelosuppression and patient-reported outcomes in patients with extensive-stage small cell lung cancer: pooled results from three phase II randomized, double-blind, placebo-controlled studies. Clin Lung Cancer (2021) 22:449–60. doi: 10.1016/j.cllc.2021.03.010 33895103

[23] Crawford J Ozer H Stoller R Johnson D Lyman G Tabbara I. Reduction by granulocyte colony-stimulating factor of fever and neutropenia induced by chemotherapy in patients with small-cell lung cancer. N Engl J Med (1991) 325:164–70. doi: 10.1056/NEJM199107183250305 1711156

[24] Sculier JP Paesmans M Lecomte J Van Cutsem O Lafitte JJ Berghmans T. A three-arm phase III randomised trial assessing, in patients with extensive-disease small-cell lung cancer, accelerated chemotherapy with support of haematological growth factor or oral antibiotics. Br J Cancer (2001) 85:1444–51. doi: 10.1054/bjoc.2001.2114 PMC2363948 11720426

[25] Weiss JM Csoszi T Maglakelidze M Hoyer RJ Beck JT Domine Gomez M. Myelopreservation with the CDK4/6 inhibitor trilaciclib in patients with small-cell lung cancer receiving first-line chemotherapy: a phase Ib/randomized phase II trial. Ann Oncol (2019) 30:1613–21. doi: 10.1093/annonc/mdz278 PMC6857609 31504118

[26] Hart LL Ferrarotto R Andric ZG Beck JT Subramanian J Radosavljevic DZ. Myelopreservation with trilaciclib in patients receiving topotecan for small cell lung cancer: results from a randomized, double-blind, placebo-controlled phase II study. Adv Ther (2021) 38:350–65. doi: 10.1007/s12325-020-01538-0 PMC7854399 33123968

[27] ClinicalTrials.gov. Carboplatin, etoposide, and atezolizumab with or without trilaciclib (G1T28), a CDK 4/6 inhibitor, in extensive stage SCLC . Available at: https://clinicaltrials.gov/ct2/show/NCT03041311 (Accessed October 13, 2021).

[28] Lara PN Jr. Moon J Redman MW Semrad TJ Kelly K Allen JW. Relevance of platinum-sensitivity status in relapsed/refractory extensive-stage small-cell lung cancer in the modern era: a patient-level analysis of southwest oncology group trials. J Thorac Oncol (2015) 10:110–5. doi: 10.1097/JTO.0000000000000385 PMC4320001 25490004

[29] Dingemans AC Fruh M Ardizzoni A Besse B Faivre-Finn C Hendriks LE. Small-cell lung cancer: ESMO Clinical Practice Guidelines for diagnosis, treatment and follow-up^☆^ . Ann Oncol (2021) 32:839–53. doi: 10.1016/j.annonc.2021.03.207 PMC9464246 33864941

[30] HYCAMTIN^®^ (topotecan). East Hanover, NJ: Novartis Pharmaceuticals (2018).

[31] TOPOTECAN injection. Sellersville, PA: Teva Pharmaceuticals (2014).

[32] Trigo J Subbiah V Besse B Moreno V López R Sala MA. Lurbinectedin as second-line treatment for patients with small-cell lung cancer: a single-arm, open-label, phase 2 basket trial. Lancet Oncol (2020) 21:645–54. doi: 10.1016/s1470-2045(20)30068-1 32224306

[33] Von Pawel J Schiller JH Shepherd FA Fields SZ Kleisbauer JP Chrysson NG. Topotecan versus cyclophosphamide, doxorubicin, and vincristine for the treatment of recurrent small-cell lung cancer. J Clin Oncol (1999) 17:658–67. doi: 10.1200/JCO.1999.17.2.658 10080612

[34] O'Brien ME Ciuleanu TE Tsekov H Shparyk Y Cucevia B Juhasz G. Phase III trial comparing supportive care alone with supportive care with oral topotecan in patients with relapsed small-cell lung cancer. J Clin Oncol (2006) 24:5441–7. doi: 10.1200/JCO.2006.06.5821 17135646

[35] Eckardt JR Von Pawel J Pujol JL Papai Z Quoix E Ardizzoni A. Phase III study of oral compared with intravenous topotecan as second-line therapy in small-cell lung cancer. J Clin Oncol (2007) 25:2086–92. doi: 10.1200/JCO.2006.08.3998 17513814

[36] ZEPZELCA^®^ (lurbinectedin). Palo Alto, CA: Jazz Pharmaceuticals, Inc (2021).

[37] Johal S Hettle R Carroll J Maguire P Wynne T. Real-world treatment patterns and outcomes in small-cell lung cancer: a systematic literature review. J Thorac Dis (2021) 13:3692–707. doi: 10.21037/jtd-20-3034 PMC8264706 34277061

[38] Baize N Monnet I Greillier L Geier M Lena H Janicot H. Carboplatin plus etoposide versus topotecan as second-line treatment for patients with sensitive relapsed small-cell lung cancer: an open-label, multicentre, randomised, phase 3 trial. Lancet Oncol (2020) 21:1224–33. doi: 10.1016/s1470-2045(20)30461-7 32888454

[39] Subbiah V Paz-Ares L Besse B Moreno V Peters S Sala MA. Antitumor activity of lurbinectedin in second-line small cell lung cancer patients who are candidates for re-challenge with the first-line treatment. Lung Cancer (2020) 150:90–6. doi: 10.1016/j.lungcan.2020.10.003 33096421

[40] Smith EF Fokkema E Biesma B Groen HJ Snoek W Postmus PE. A phase II study of paclitaxel in heavily pretreated patients with small-cell lung cancer. Br J Cancer (1998) 77:347–51. doi: 10.1038/bjc.1998.54 PMC2151229 9461009

[41] Yamamoto N Tsurutani J Yoshimura N Asai G Moriyama A Nakagawa K. Phase II study of weekly paclitaxel for relapsed and refractory small cell lung cancer. Anticancer Res (2006) 26:777–81. 16739353

[42] Smyth JF Smith IE Sessa C Schoffski P Wanders J Franklin H. Activity of docetaxel (Taxotere) in small cell lung cancer. The Early Clinical Trials Group of the EORTC. Eur J Cancer (1994) 30A:1058–60. doi: 10.1016/0959-8049(94)90455-3 7654428

[43] Masuda N Fukuoka M Kusunoki Y Matsui K Takifuji N Kudoh S. CPT-11: a new derivative of camptothecin for the treatment of refractory or relapsed small-cell lung cancer. J Clin Oncol (1992) 10:1225–9. doi: 10.1200/JCO.1992.10.8.1225 1321891

[44] Pietanza MC Kadota K Huberman K Sima CS Fiore JJ Sumner DK. Phase II trial of temozolomide in patients with relapsed sensitive or refractory small cell lung cancer, with assessment of methylguanine-DNA methyltransferase as a potential biomarker. Clin Cancer Res (2012) 18:1138–45. doi: 10.1158/1078-0432.CCR-11-2059 22228633

[45] Zauderer MG Drilon A Kadota K Huberman K Sima CS Bergagnini I. Trial of a 5-day dosing regimen of temozolomide in patients with relapsed small cell lung cancers with assessment of methylguanine-DNA methyltransferase. Lung Cancer (2014) 86:237–40. doi: 10.1016/j.lungcan.2014.08.007 PMC4497567 25194640

[46] Johnson JR Williams G Pazdur R. End points and United States Food and Drug Administration approval of oncology drugs. J Clin Oncol (2003) 21:1404–11. doi: 10.1200/JCO.2003.08.072 12663734

[47] Jassem J Karnicka-Mlodkowska H Van Pottelsberghe C Van Glabbeke M Noseda MA Ardizzoni A. Phase II study of vinorelbine (Navelbine) in previously treated small cell lung cancer patients. EORTC Lung Cancer Cooperative Group. Eur J Cancer (1993) 29A:1720–2. doi: 10.1016/0959-8049(93)90112-s 8398301

[48] Furuse K Kubota K Kawahara M Takada M Kimura I Fujii M. Phase II study of vinorelbine in heavily previously treated small cell lung cancer. Japan Lung Cancer Vinorelbine Study Group. Oncology (1996) 53:169–72. doi: 10.1159/000227555 8604245

[49] Van Der Lee I Smit EF Van Putten JW Groen HJ Schlosser NJ Postmus PE. Single-agent gemcitabine in patients with resistant small-cell lung cancer. Ann Oncol (2001) 12:557–61. doi: 10.1023/a:1011104509759 11398892

[50] Masters GA Declerck L Blanke C Sandler A Devore R Miller K. Phase II trial of gemcitabine in refractory or relapsed small-cell lung cancer: Eastern Cooperative Oncology Group Trial 1597. J Clin Oncol (2003) 21:1550–5. doi: 10.1200/JCO.2003.09.130 12697880

[51] Antonia SJ López-Martin JA Bendell J Ott PA Taylor M Eder JP. Nivolumab alone and nivolumab plus ipilimumab in recurrent small-cell lung cancer (CheckMate 032): a multicentre, open-label, phase 1/2 trial. Lancet Oncol (2016) 17:883–95. doi: 10.1016/s1470-2045(16)30098-5 27269741

[52] Ready NE Ott PA Hellmann MD Zugazagoitia J Hann CL De Braud F. Nivolumab monotherapy and nivolumab plus ipilimumab in recurrent small cell lung cancer: results from the CheckMate 032 randomized cohort. J Thorac Oncol (2020) 15:426–35. doi: 10.1016/j.jtho.2019.10.004 31629915

[53] Ott PA Elez E Hiret S Kim DW Morosky A Saraf S. Pembrolizumab in patients with extensive-stage small-cell lung cancer: results from the phase Ib KEYNOTE-028 study. J Clin Oncol (2017) 35:3823–9. doi: 10.1200/JCO.2017.72.5069 28813164

[54] Chung HC Lopez-Martin JA Kao SC Miller WH Ros W Gao B. Phase 2 study of pembrolizumab in advanced small-cell lung cancer (SCLC): KEYNOTE-158. J Clin Oncol (2018) 36:8506. doi: 10.1200/JCO.2018.36.15_suppl.8506

[55] Chung HC Piha-Paul SA Lopez-Martin J Schellens JHM Kao S Miller WH Jr.. Pembrolizumab after two or more lines of previous therapy in patients with recurrent or metastatic SCLC: results from the KEYNOTE-028 and KEYNOTE-158 Studies. J Thorac Oncol (2020) 15:618–27. doi: 10.1016/j.jtho.2019.12.109 31870883

[56] Lammers PE Shyr Y Li CI Hutchison AS Sandler A Carbone DP. Phase II study of bendamustine in relapsed chemotherapy sensitive or resistant small-cell lung cancer. J Thorac Oncol (2014) 9:559–62. doi: 10.1097/JTO.0000000000000079 PMC3990869 24736081

[57] Pharmamar. PharmaMar and Jazz Pharmaceuticals announce initiation of confirmatory phase III clinical trial of Zepzelca^®^ (lurbinectedin) for the treatment of patients with relapsed small cell lung cancer (2021). Available at: https://pharmamar.com/en/pharmamar-and-jazz-pharmaceuticals-announce-initiation-of-confirmatory-phase-iii-clinical-trial-of-zepzelca-lurbinectedin-for-the-treatment-of-patients-with-relapsed-small-cell-lung-cancer/ (Accessed January 13, 2022).

[58] Pharmamar. Clinical trial of lurbinectedin as single-agent or in combination with irinotecan versus topotecan or irinotecan in patients with relapsed small-cell lung cancer (LAGOON). ClinicalTrials.gov. Available at: https://www.clinicaltrials.gov/ct2/show/NCT05153239 (Accessed January 23, 2022).

[59] Paz-Ares L Ciuleanu TE Navarro A Ciuleanu TE Navarro A Fulop A. Combination lurbinectedin and doxorubicin versus physician's choice of chemotherapy in patients with relapsed small-cell lung cancer (ATLANTIS): a multicentre, randomised, open-label, phase 3 trial. Lancet Respir Med (2023) 11(1):74–86. doi: 10.1016/S2213-2600(22)00309-5 36252599

[60] Jazz Pharmaceuticals. Jazz Pharmaceuticals and PharmaMar announce results of ATLANTIS phase 3 study evaluating Zepzelca™ in combination with doxorubicin for patients with small cell lung cancer following one prior platinum-containing line. Available at: https://investor.jazzpharma.com/news-releases/news-release-details/jazz-pharmaceuticals-and-pharmamar-announce-results-atlantis (Accessed January 23, 2023).

[61] ABRAXANE^®^ for injectable suspension (paclitaxel protein-bound particles for injectable suspension) (albumin-bound). Summit, NJ: Celgene Corporation (2013).

[62] TAXOL^®^ (paclitaxel) injection. Princeton, NJ: Bristol Myers Squibb Company (2011).

[63] CAMPTOSAR^®^ (irinotecan). New York, NY: Pharmacia & Upjohn Co (2014).

[64] Stupp R Hegi ME Mason WP Van Den Bent MJ Taphoorn MJ Janzer RC. Effects of radiotherapy with concomitant and adjuvant temozolomide versus radiotherapy alone on survival in glioblastoma in a randomised phase III study: 5-year analysis of the EORTC-NCIC trial. Lancet Oncol (2009) 10:459–66. doi: 10.1016/S1470-2045(09)70025-7 19269895

[65] Ready N Farago AF De Braud F Atmaca A Hellmann MD Schneider JG. Third-line nivolumab monotherapy in recurrent SCLC: CheckMate 032. J Thorac Oncol (2019) 14:237–44. doi: 10.1016/j.jtho.2018.10.003 PMC8050700 30316010

[66] Wong SK Iams WT. Front line applications and future directions of immunotherapy in small-cell lung cancer. Cancers (Basel) (2021) 13:506. doi: 10.3390/cancers13030506 33572705 PMC7865814

[67] Owonikoko TK Borghaei H Champiat S Paz-Ares LG Govindan R Boyer MJ. Phase I study of AMG 757, a half-life extended bispecific T-cell engager (HLE BiTE immune therapy) targeting DLL3, in patients with small cell lung cancer (SCLC). J Clin Oncol (2020) 38:TPS9080. doi: 10.1200/JCO.2020.38.15_suppl.TPS9080

[68] Giffin MJ Cooke K Lobenhofer EK Estrada J Zhan J Deegen P. AMG 757, a half-life extended, DLL3-targeted bispecific T-cell engager, shows high potency and sensitivity in preclinical models of small-cell lung cancer. Clin Cancer Res (2021) 27:1526–37. doi: 10.1158/1078-0432.CCR-20-2845 33203642

[69] Owonikoko TK Champiat S Johnson ML Govindan R Izumi H Lai WVV. Updated results from a phase 1 study of AMG 757, a half-life extended bispecific T-cell engager (BiTE) immuno-oncology therapy against delta-like ligand 3 (DLL3), in small cell lung cancer (SCLC). J Clin Oncol (2021) 39:8510. doi: 10.1200/JCO.2021.39.15_suppl.8510

[70] National Institutes of Health. Phase 3 study of pexidartinib for pigmented villonodular synovitis (PVNS) or giant cell tumor of the tendon sheath (GCT-TS) (ENLIVEN) . Available at: https://clinicaltrials.gov/ct2/show/NCT02371369 (Accessed January 23, 2023).

[71] Aaron W Austin R Barath M Callihan E Cremin M Evans T. HPN328: An anti-DLL3 T cell engager for treatment of small cell lung cancer. Mol Cancer Ther (2019) 18(suppl 12):C033. doi: 10.1158/1535-7163.TARG-19-C033

[72] Clinicaltrials.Gov. Study in patients with advanced cancers associated with expression of DLL3 who have failed standard available therapy (2020). Available at: https://clinicaltrials.gov/ct2/show/NCT04471727 (Accessed September 1, 2021).

[73] Pietanza MC Waqar SN Krug LM Dowlati A Hann CL Chiappori A. Randomized, double-blind, phase II study of temozolomide in combination with either veliparib or placebo in patients with relapsed-sensitive or refractory small-cell lung cancer. J Clin Oncol (2018) 36:2386–94. doi: 10.1200/JCO.2018.77.7672 PMC6085179 29906251

[74] Zhang B Stewart CA Gay CM Wang Q Cardnell R Fujimoto J. Detection of DNA replication blocker SLFN11 in tumor tissue and circulating tumor cells to predict platinum response in small cell lung cancer. Cancer Res (2021) 18(suppl 13):384. doi: 10.1158/1538-7445.Am2021-384

[75] ClinicalTrials.gov. Testing maintenance therapy for small cell lung cancer in patients with SLFN11 positive biomarker (2020). Available at: https://clinicaltrials.gov/ct2/show/NCT04334941 (Accessed January 13, 2022).

[76] ClinicalTrials.gov. Study of irinotecan liposome injection (ONIVYDE^®^) in patients with small cell lung cancer (RESILIENT) (2017). Available at: https://clinicaltrials.gov/ct2/show/NCT03088813 (Accessed September 1, 2021).

[77] Paz-Ares LG Spigel DR Chen Y Maria Jove M Juan-Vidal O Rich P. RESILIENT part II: an open-label, randomized, phase III study of liposomal irinotecan injection in patients with small-cell lung cancer who have progressed with platinum-based first-line therapy. J Clin Oncol (2020) 38:TPS9081. doi: 10.1200/JCO.2020.38.15_suppl.TPS9081

[78] Gay CM Stewart CA Park EM Diao L Groves SM Heeke S. Patterns of transcription factor programs and immune pathway activation define four major subtypes of SCLC with distinct therapeutic vulnerabilities. Cancer Cell (2021) 39:346–360.e7. doi: 10.1016/j.ccell.2020.12.014 33482121 PMC8143037

[79] Rudin CM Poirier JT Byers LA Dive C Dowlati A George J. Molecular subtypes of small cell lung cancer: a synthesis of human and mouse model data. Nat Rev Cancer (2019) 19:289–97. doi: 10.1038/s41568-019-0133-9 PMC6538259 30926931

[80] Wagner AH Devarakonda S Skidmore ZL Krysiak K Ramu A Trani L. Recurrent WNT pathway alterations are frequent in relapsed small cell lung cancer. Nat Commun (2018) 9:3787. doi: 10.1038/s41467-018-06162-9 30224629 PMC6141466

[81] Mahadevan NR Knelson EH Wolff JO Vajdi A Saigi M Campisi M. Intrinsic immunogenicity of small cell lung carcinoma revealed by its cellular plasticity. Cancer Discovery (2021) 11:1952–69. doi: 10.1158/2159-8290.CD-20-0913 PMC8338750 33707236

[82] Byers LA Rudin CM. Small cell lung cancer: where do we go from here? Cancer (2015) 121:664–72. doi: 10.1002/cncr.29098 PMC5497465 25336398

